# Composite Cationic Exchange Membranes Prepared from Polyvinyl Alcohol (PVA) and Boronic Acid Copolymers for Alkaline Diffusion Dialysis

**DOI:** 10.3390/ma11081354

**Published:** 2018-08-04

**Authors:** Dandan Liu, Congwei Wang, Jibin Miao, Ru Xia, Peng Chen, Ming Cao, Bin Wu, Jiasheng Qian

**Affiliations:** Anhui Province Key Laboratory of Environment-friendly Polymer Materials, School of Chemistry & Chemical Engineering, Anhui University, Hefei 230601, China; 18756563397@163.com (D.L.); wcw215@126.com (C.W.); xiarucn@sina.com (R.X.); chpecp@126.com (P.C.); cmcmycyc@163.com (M.C.); 17705@ahu.edu.cn (B.W.)

**Keywords:** polyvinyl alcohol (PVA), boronic acid copolymers, composite membranes, diffusion dialysis (DD), alkali recovery

## Abstract

Boronic acid copolymers with a large number of functional groups were obtained via a free radical polymerization method. The corresponding composite membranes for alkaline diffusion dialysis (DD) were prepared by a crosslinking reaction between polyvinyl alcohol (PVA) and the as-obtained boronic acid copolymers. The composite membranes possessed water uptake (W_R_) of 122.1–194.4%, ion exchange capacities (IECs) of 0.79–1.17 mmol/g, as well as both good mechanical stability (tensile strength of 40.7–49.9 MPa and elongation at break of 52.1–121.1%) and thermal stability. The as-prepared membranes were stable in 2 M NaOH solution at 65 °C with a swelling degree and mass loss of as-prepared membranes in the range of 305–368% and 9.2–22%, respectively. A NaOH/Na_2_WO_4_ mixture was used to investigate the separation properties of as-prepared membranes via the DD process. Results indicated that the dialysis coefficients of OH^−^ (U_OH_) were in the range of 0.0079–0.0150 m/h, while the separation factors (*S*) were in the range of 26.6–53.2. The functional groups from boronic acid copolymer and –OH from PVA were demonstrated to promote the ion transport synergistically.

## 1. Introduction

The diffusion dialysis (DD) process provides an environmentally friendly way to treat alkaline waste water. Compared with traditional methods, the DD process exhibits lower cost and energy consumption [1]. There has been much research concentrating on the acidic DD process and anion exchange membranes for commercial use (e.g., DF120 with U_H_^+^ of 0.00136 m/h and separation factor (*S*) of 36.3) [1]. On the contrary, membranes for alkali recovery have been infrequently reported. The membrane is the core component of the DD process. Therefore, more and more researchers are focusing their attention on the research and development of novel membrane materials for the alkaline DD process. Considering that most polymers with strong-base anion-exchange groups are unstable in alkaline environment, only a few polymer membranes can be used in the alkaline DD process. In the reported researches, sulfonated poly (2,6-dimethyl-1,4-phenyleneoxide) (SPPO) [2,3,4,5], polyvinyl alcohol (PVA) [6,7,8], and other polymers-based membranes [9,10] were successfully used for alkaline DD. The as-prepared membranes possessed a certain character in ion flux (e.g., PVA) [6,7,8], ion selectivity (e.g., SPPO) [2,3,4,5] or alkali resistance (e.g., chlorosulfonated polyethylene (CSM) [10] and polyvinylidene fluoride (PVDF) [9]).

Among these polymer materials, PVA has attracted remarkable attention due to its good membrane-forming ability as well as hydrophilicity. However, in addition, it is necessary for PVA-based membranes to incorporate functional groups (such as –COO^−^ or –SO_3_^−^) and crosslink linear PVA chains to enhance sustainability during the running process. For instance, Yonghui Wu et al. [11,12,13] prepared a series of PVA-based organic–inorganic hybrid membranes from a PVA solution and a multi-silicon crosslinking agent via the sol-gel process. The as-prepared hybrid membranes possessed enhanced ion flux as well as OH^−^ ion selectivity compared with the commercial ones. The authors concluded that functional groups (e.g., –SO_3_^−^ and –COO^−^) from the multi-silicon crosslinking agent and –OH from PVA promoted ion transport synergistically. Therefore, it was implied that polymeric crosslinking agents for PVA could be a good strategy to prepare membranes for alkaline DD. However, organic solvents used during the synthesis of multi-silicon crosslinking agent are usually non-eco-friendly. In addition, O–Si–O bonds tend to break under the immersion of strong base, which could negate the separation performance of the hybrid membranes. Therefore, it was deemed necessary to develop new methods to prepare novel and eco-friendly crosslinking agents.

Boronic acid can react with PVA under pH value of 8–9 but the reaction is usually difficult to control [14]. In view of the problem, boronic acid copolymers with a large number of functional groups were synthesized and were selected as crosslinking agents to prepare composite membranes for alkaline DD. The as-prepared membranes possessed the following advantages: first, introduction of boronic acid copolymer endowed the membrane with many functional groups and sustained the flexibility of the PVA chain [15]; second, the crosslinking reaction rate between PVA and boronic acid copolymers could be more controllable [16,17]; finally, all the reactions, including the synthesis of the boronic acid copolymers and the formation of composite membranes, were carried out in aqueous solutions without any organic solvent. The relationship between physicochemical properties and separation performance of as-prepared membranes is discussed preliminarily.

## 2. Experimental

### 2.1. Materials

PVA, NaOH, HCl, Na_2_WO_4_, (NH4)_2_S_2_O_8_ (APS) and NaHSO_3_ (SHS) with analytical purity were purchased from Sinopharm Chemical Regent Co., Ltd. (Shanghai, China). (3-acrylamido)phenylboronic acid (AAPBA) and 2-acrylamide-2-methyl propane sulfonic acid (AMPS) with analytical purity were purchased from Boron Nobel Technology Co., Ltd. (Beijing, China). PVA was dissolved in deionized water to form a 5% concentration solution as in previous reports [13]. The feed solution of NaOH (1.0 mol/L)/Na_2_WO_4_ (0.10 mol/L) was prepared freshly [18] and deionized (DI) water was utilized throughout.

### 2.2. Synthesis of Boronic Acid Copolymers

**Scheme 1:**
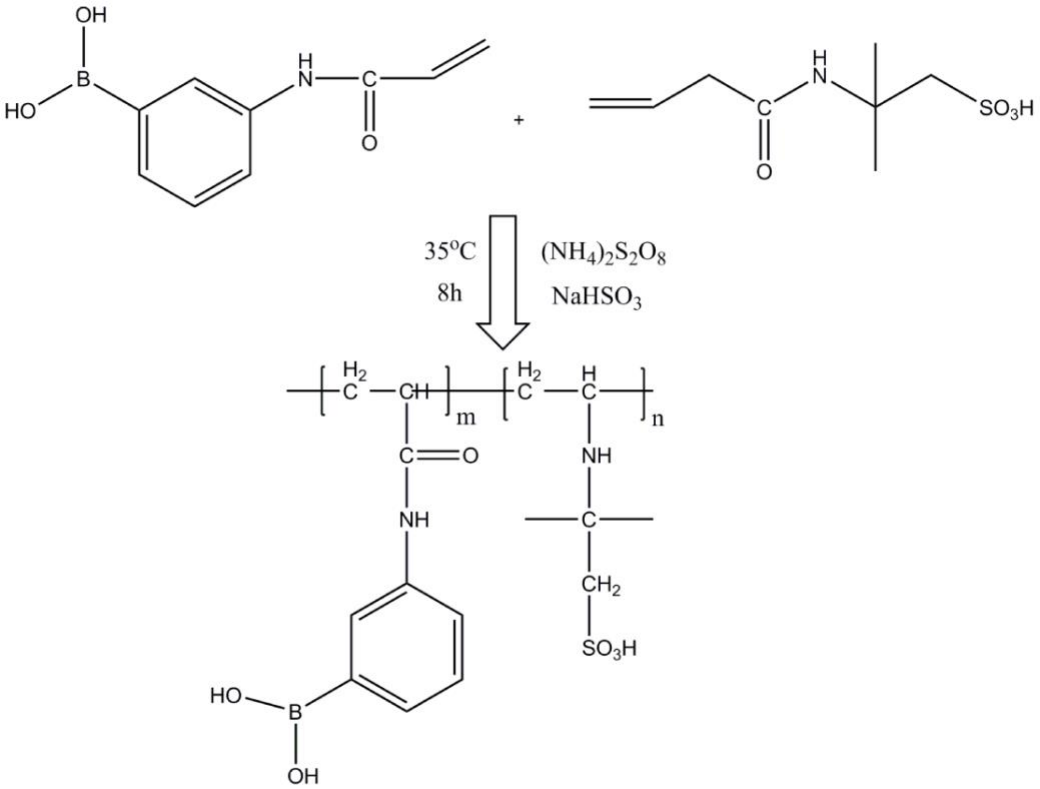
Schematic diagram for the synthesis of boronic acid copolymer.

In a typical synthesis process, 2.07 g of 2-acrylamide-2-methyl propane sulfonic acid (AMPS) and 0.19 g of (3-acrylamido)phenylboronic acid (AAPBA) were dissolved in 60 mL of water to form a transparent solution. After that, a mixture of 13.6 mg of APS and 4.52 mg of SHS was added into the above solution under continuous stirring (mass ratio of initiator to total monomers was 0.8%) at room temperature. An aqueous solution of boronic acid copolymers was obtained after the mixture was kept at 35 °C for 8 h. The reaction process is displayed in Scheme 1.

### 2.3. Preparation of Composite Cationic Membranes

Various amounts of boronic acid copolymers with mass ratios of boronic acid copolymers to PVA 0%, 1%, 2%, 4%, and 8%, respectively—the PVA solution became too viscous to form a membrane when dosage of boronic acid copolymer was higher than 8%—were added dropwise to 40 mL of PVA solution under vigorous stirring. The pH value of the system was controlled as 8–9 and the reaction time was set as 30 min at 35 °C. The obtained solution was cast onto a clean glass plate (20 cm × 20 cm) and dried in the air at room temperature. After the solvent evaporated completely, the membranes were peeled and heated from 70 to 100 °C at a heating rate of 10 °C/h. The as-prepared membranes were marked as 0%, 1%, 2%, 4%, and 8%, respectively.

### 2.4. Characterization Methods and Diffusion Dialysis (DD) Testing

The Fourier transform infrared (FTIR) spectrum of boronic acid copolymer was taken by a Nicolet iS10 spectrometer (Thermo Fisher Scientific, Waltham, MA, USA) at a scanning range of 4000–400 cm^−1^. Ion exchange capacities (IECs), water uptake (W_R_), and alkali resistance of as-prepared membranes were tested as reported in our previous works and two duplicate samples were tested simultaneously [10]. The mechanical stability of as-prepared membranes was taken on a CMT6104 (MTS Industrial System, China CO., Ltd., Shenzhen, China) universal testing machine and three duplicate samples were tested for each membrane. Thermal stability was tested by a Shimadzu TGA-50H analyzer (Shimadzu, Kyoto, Japan) under air flow from 25 to 700 °C. Microscopic morphologies of as-prepared membranes were recorded by a scanning electron microscopy (S-4800, Hitachi Limited, Tokyo, Japan) and coated with gold before tests.

The DD process of as-prepared membranes was tested as described in our previous reports [2,10]: The two compartments were maintained in thermostatic conditions and stirred at an identical rate to minimize the concentration polarization. The effective area of membranes was 6 cm^2^ and the DD process was enabled for 1 h. The calculation of dialysis coefficients (U) and separation factors (*S*) was as follows (Equation (1)):

(1) $$U=\frac{M}{\mathrm{At}\Delta C}\;$$

where M was the amount of component transported in moles, A was the effective area in square meters, t was the time in hours, and ΔC was the logarithmic mean of the concentration difference between the two chambers as in Equation (2):

(2) $$\Delta C=\frac{C_{f}^{0}{-(C}_{f}^{t}{-C}_{d}^{t})}{{\ln[C}_{f}^{0}{/(C}_{f}^{t}{-C}_{d}^{t})]}$$

where $C_{f}^{0}$ and $C_{f}^{t}$ were the feed concentrations at time 0 and t, respectively and $C_{d}^{t}$ was the dialysate concentration at time t. The separation factor (*S*) was given as the ratio of dialysis coefficients (U) of OH^−^ to that of WO_4_^2−^.

## 3. Results and Discussion

### 3.1. FT-IR Spectrum of Boronic Acid Copolymer

**Figure 1:**
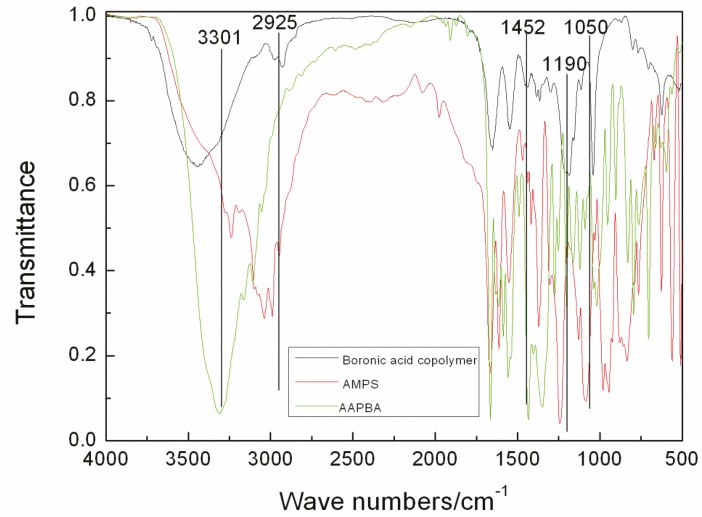
Fourier transform infrared (FTIR) spectrum of boronic acid copolymer.

The FT-IR spectrum of boronic acid copolymer is shown in Figure 1. The absorption peaks at 3301 cm^−1^ and 1452 cm^−1^ correspond to the stretching vibration and bending vibration of –OH, respectively, which indicates that the sample contains a large number of hydroxyl groups. The strong adsorption peaks at 1190 cm^−1^ and 1050 cm^−1^ confirm the existence of –SO_3_^−^. Disappearance of the adsorption band at 3075–3090 cm^−1^ confirms the breakage of the C=C bond while the appearance of the adsorption band at 2925 cm^−1^ indicates the successful synthesis of boronic acid copolymers [19].

### 3.2. Water Uptake (W_R_) and Ion Exchange Capacities (IECs)

The water uptake performance of as-prepared membranes is shown in Table 1, which is in the range of 122.1%–194.4%. Water uptake of the membrane is from two aspects: the hydrophilic groups –SO_3_^−^ and –OH and the space between PVA chains. The W_R_ of PVA decreased slightly when the dosage of boronic acid copolymer was 1% due to a decrease of –OH. On further increasing the dosage of boronic acid copolymer to 2%, the W_R_ of the membrane reached the maximum value, which was due to the synergistic effect between –SO_3_^−^ and –OH. Even though –SO_3_^−^ exhibited stronger hydrophilicity than –OH [20], the resulting decrease of W_R_ for the 4% and 8% samples was attributed to the lower –OH content and increased density of the composite membranes.

The IECs of composite membranes are shown in Table 1, which are comparable with those of previous reports [6,7,8]. The IEC was difficult to obtain when the dosage of boronic acid copolymer was smaller than 1% and mainly due to the low content of functional groups. The 4% sample exhibited the maximum IEC value (1.17 ± 0.01) and that of the 8% sample decreased to 1.05 ± 0.01, which can be attributed to the difficult exchange of functional groups with a higher crosslinking degree. Considering the ion transport and stability, membranes used in the DD process need restricted W_R_ and moderate IECs [1]. The results indicate that membranes in this study are suitable for the DD process.

### 3.3. Alkali Resistance

Alkali resistance results of as-prepared membranes are shown in Table 2. The swelling degrees of composite membranes were higher than that of pure PVA, indicating that the incorporation of boronic acid copolymers facilitated the attack of OH^−^ on the membrane matrix. The increasing trend climbed gently with increased dosage of boronic acid copolymer due to the larger crosslinking degree between –OH and –B(OH)_2_.

The mass loss of composite membranes was mainly derived from the degradation of crosslinked polymer chains [8]. Compared with previous reports based on PVA matrix [6,7,8], the mass loss of composite membranes in this work was acceptable, which was only 9.2% when the dosage of boronic acid copolymer was 4%.

When the heating temperature increased to 120 °C, the swelling degree of membranes was restricted due to the enhanced density of the as-prepared membranes. By contrast, increasing the heating temperature cannot actually prevent membranes from attack of OH^−^. The results indicated that the factors contributing to the alkali resistance of as-prepared membranes were complicated.

### 3.4. Mechanical Properties of As-Prepared Membranes

Mechanical properties including tensile strength (TS) and elongation at break (E_b_) of membranes heated at 100 °C and 120 °C are presented in Table 3, from which we can conclude that membranes heated at 100 °C exhibit better mechanical properties. For membranes heated at 100 °C, the TS and E_b_ of all the samples are in the range of 40.7–49.9 MPa and 52.1–121.1%, respectively. The TS of the membranes is higher than those of previous PVA-based ion exchangeable membranes but exhibits similar trends [13]. The reasons might be as follows: The addition of boronic acid copolymer enhanced the membrane crosslinking degree and thus TS increased first; excessive content of boronic acid copolymer affected the aggregation of the PVA main chains and thus the TS decreased when the dosage of boronic acid copolymer was higher than 2%. The E_b_ of as-prepared membranes decreased obviously with increased dosage of boronic acid copolymer, which was due to the rearrangement of PVA chains based on the interaction between hydrophilic –OH and –SO_3_^−^ [18]. The membranes exhibited optimal mechanical properties with TS and E_b_ of 49.9 MPa and 87.0%, respectively when the dosage of boronic acid copolymer was 2%.

### 3.5. Thermal Stability Results of As-Prepared Membranes

**Figure 2:**
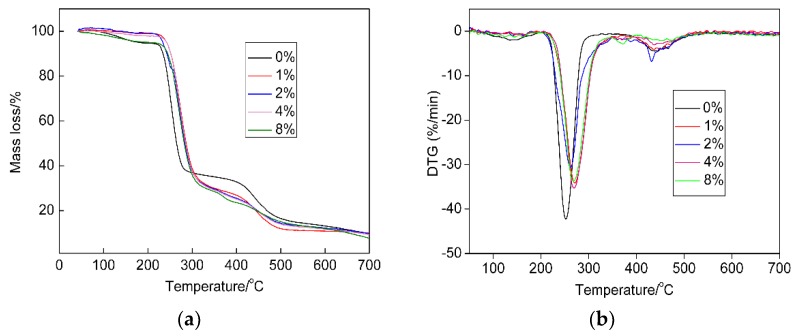
TG and DTG curves of as-prepared membranes (after 100 °C treatment). (**a**) TG curves; (**b**) DTG curves.

Thermogravimetric (TG) and differential thermogravimetric (DTG) curves of as-prepared membranes are shown in Figure 2 and two mass loss slopes of each sample are observed. The mass loss slope in the range from 200 to 300 °C corresponds to the degradation of OH^−^ and –SO_3_^−^, in which the obvious delayed trend could be due to the formation of a crosslinking structure between –OH and B(OH)_2_. The results indicate that the dosage of boronic acid copolymers is favorable to promote the thermal stability of functional groups. The second mass loss slope between 400 °C and 500 °C corresponds to the degradation of the PVA main chains, suggesting that the incorporation of boronic acid copolymers has no significant effect on the thermal stability of the carbon chains.

### 3.6. Microscopic Morphologies of As-Prepared Membranes

**Figure 3:**
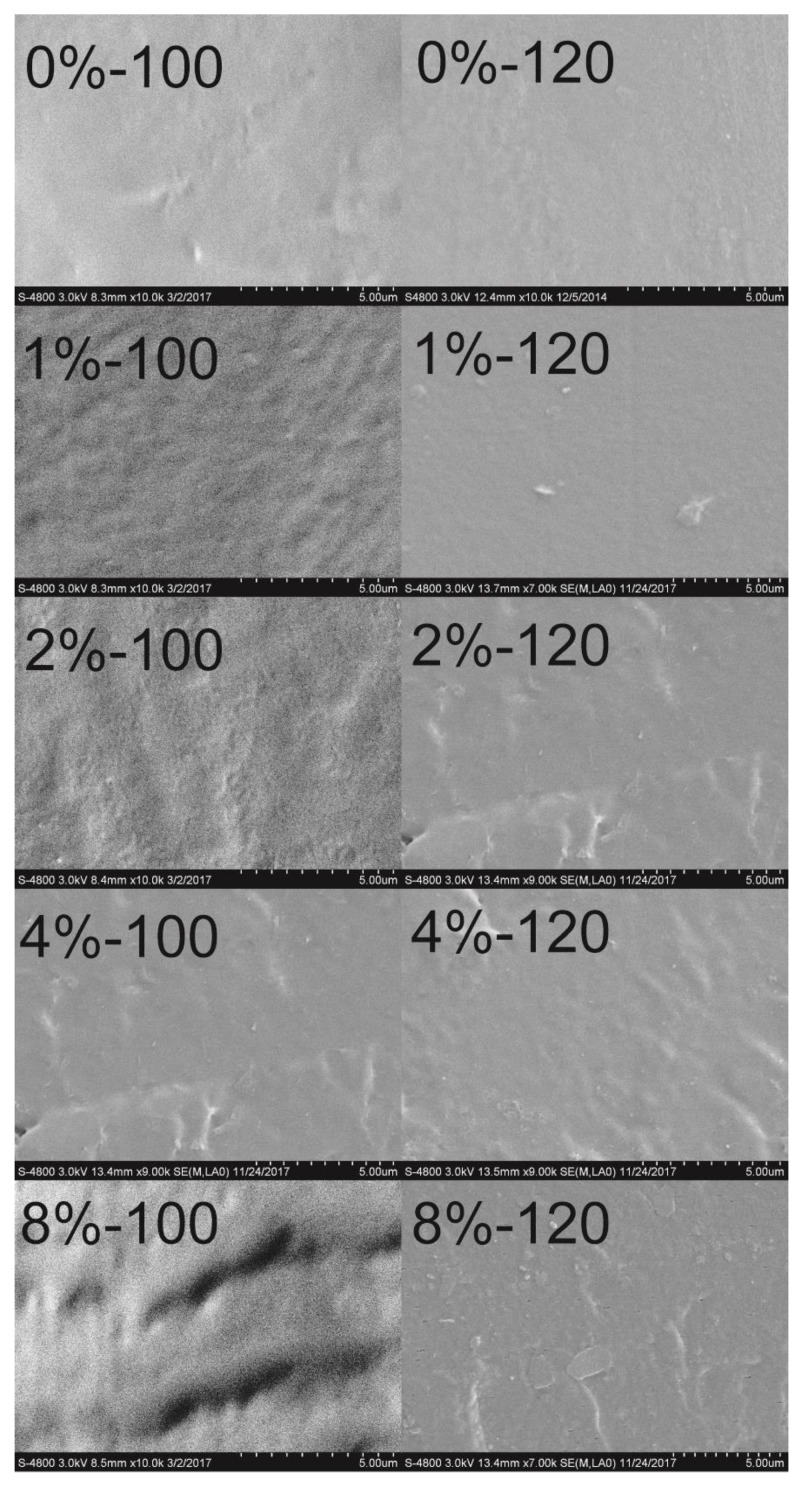
Cross section scanning electron microscopy (SEM) pictures of as-prepared membranes (after thermal treatment at 100 °C and 120 °C, respectively. Naming of samples: x%-y, where x was the dosage of boronic acid copolymer and y was the heating temperature).

Cross section scanning electron microscopy (SEM) images of as-prepared membranes are shown in Figure 3. Incorporation of boronic acid copolymer exhibited no significant effect on the cross-section morphologies of as-prepared membranes when the dosage of boronic acid copolymer was less than 8%, revealing good compatibility between the two phases. However, an obvious structure fault emerged when the dosage of boronic acid copolymer increased continuously to 8%. The dense structure ensured the possible application in the alkaline DD process of the as-prepared membranes.

In Figure 3, obvious caves could be observed with increasing dosage of boronic acid copolymers, especially in the designated membrane 8%-120. This could be due to the fact that the combination between PVA and boronic acid copolymer by physical crosslinking interaction may have been interrupted at higher temperatures. Thus, structural caves were formed and the separation performance of the composite membranes decreased.

### 3.7. Diffusion Dialysis (DD) Results

**Figure 4:**
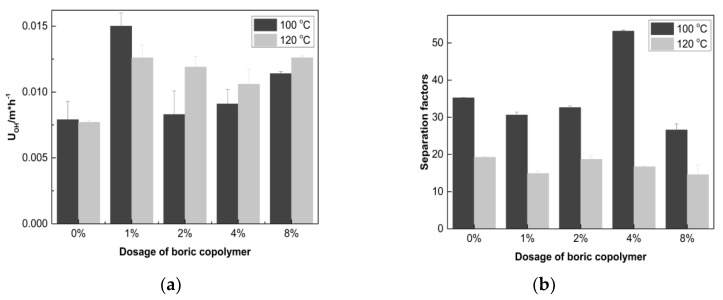
Diffusion dialysis (DD) results of composite membranes after heating at 100 °C and 120 °C, respectively. (**a**) Dialysis coefficient; (**b**) separation factors.

In the previous researches [5,8,11,12,13], PVA-based composite membranes were usually treated at a high temperature to promote the sol-gel process as well as the condensation between –OH groups. Therefore in this work, composite membranes were heated at 100 °C and 120 °C to detect the effects of thermal treatment conditions on the separation performance of the composite membranes. Dialysis coefficient (U_OH_) and separation factors (*S*) of the two groups in the composite membranes are shown in Figure 4. The separation factors of the composite membranes decreased rapidly when the thermal treatment temperature was 120 °C, which was different from previous researches [5,8]. The reason for this was the formation of caves in the membranes matrix when the heating temperature increased.

**Figure 5:**
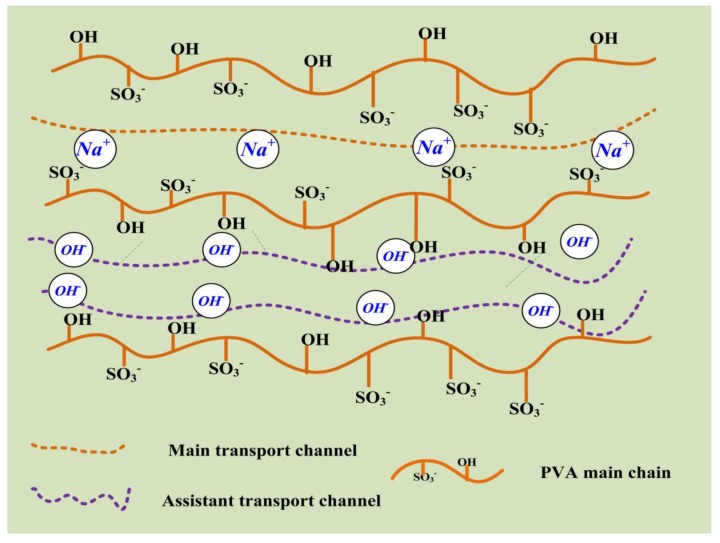
Mimetic transport process of Na^+^ and OH^−^.

Composite membranes exhibited higher U_OH_ than the counterpart under the two thermal treatment temperatures, which confirmed that the incorporation of boronic acid copolymer was advantageous to enhance OH^−^ transport. The composite membrane with 2% of boronic acid copolymer exhibited the highest U_OH_ value. Considering the decreasing –OH amount under thermal treatment, the DD results indicated that both functional groups (–SO_3_^−^) and assisted groups (–OH) were significant for the transport of OH^−^. The ion transport process could be explained as follows: functional groups (–SO_3_^−^) form main channels for Na^+^ via electrostatic attraction while –OH provides assisted channels for OH^−^ via hydrogen bonds. The mimetic transport process of Na^+^ and OH^−^ is illustrated in Figure 5 and this is in agreement with our previous report [2].

From the results we can conclude: (1) All the composite membranes exhibited higher U_OH_ than that of pure PVA, which confirmed the advantages of incorporation of boronic acid copolymers; (2) Compared with membranes that were heated at 100 °C, membranes after heat-treatment at 120 °C exhibited lower ion selectivity with *S* values less than 20. The results indicated that the heating temperature had a significant effect on the selectivity of as-prepared composite membranes.

The DD performance of as-prepared membranes could be evaluated by comparison with previously reported research, of which U_OH_ and S are summarized in Table 4. From Table 4, we can conclude that:

(1) The U_OH_ of as-prepared membranes was higher than that of SPPO, CSM or PVDF based membranes, which was due to the hydrophilic nature of the PVA matrix. The results indicated that incorporation of boronic acid copolymers to the PVA matrix was favorable to the transport of OH^−^.

(2) The *S* of as-prepared membranes was comparable with those of PVA-based membranes. The moderate ability of ion selectivity of as-prepared membranes was due to the easy-swelling nature of PVA. *S* reaching the maximum value when the dosage of boronic acid copolymer was 4%.

The results of the separation factors confirmed the mimetic transport process mentioned above. The U_OH_ and separation factor of the composite membranes were enhanced synchronously when the dosage of boronic acid copolymer was 4%, which showed that the incorporation of boronic acid copolymers could be one of the candidates to solve the “tradeoff effects” between ion flux and selectivity.

## 4. Conclusions

A series of composite membranes was prepared by addition of boronic acid copolymers into PVA solution. Water uptake (W_R_), ion exchange capacities (IECs), alkali resistance, microscopic morphologies, and separation performance of the as-prepared composite membranes were investigated. The results indicated that the U_OH_ (0.0091 m·h^−1^) and separation factor (53.2) were enhanced synchronously under the optimal membrane-formation conditions when the dosage of boronic acid copolymer was 4% and the thermal treatment temperature was 100 °C. The anions –SO_3_^−^ and –OH were both considered to play important roles synergistically on the ion transport: –SO_3_^−^ formed main ion channels for Na^+^ via electrostatic attraction while –OH provided assisted ion channels for OH^−^ via hydrogen bonds.

## Figures and Tables

**Table 1:** Water uptake (W_R_) and ion exchange capacity of membranes (after 100 °C treatment).

Dosage of Crosslinking Agent	W_R_ (%)	IEC (mmol/g)
0%	136.0 ± 1.6	/
1%	122.1 ± 2.2	0.79 ± 0.01
2%	194.4 ± 0.5	1.07 ± 0.02
4%	146.3 ± 0.9	1.17 ± 0.01
8%	145.4 ± 1.3	1.05 ± 0.01

**Table 2:** Alkali resistance results of composite membranes (after heating at 100 °C and 120 °C, respectively).

Dosage of Crosslinking Agent	Swelling Degree (%) 100 °C/120 °C	Mass Loss (%) 100 °C/120 °C
0%	305 ± 3/265 ± 3	11.6 ± 0.3/9.1 ± 0.3
1%	316 ± 5/301 ± 5	22.0 ± 1.1/22.0 ± 1.4
2%	368 ± 7/324 ± 4	10.0 ± 1.1/26.0 ± 0.5
4%	358 ± 4/312 ± 3	9.20 ± 0.4/6.8 ± 0.4
8%	371 ± 3/331 ± 3	16.6 ± 2.1/18.0 ± 0.8

**Table 3:** Mechanical properties of as-prepared membranes (after heating at 100 °C and 120 °C, respectively; dry).

Dosage of Boric Acid Copolymer	TS (MPa) 100 °C/120 °C	E_b_ (%) 100 °C/120 °C
0%	46.3 ± 3.2/19.2 ± 2.8	121.1 ± 7.4/153.1 ± 6.8
1%	46.8 ± 4.2/20.8 ± 3.4	88.1 ± 5.5/172.6 ± 6.2
2%	49.9 ± 4.3/16.4 ± 2.6	87.0 ± 8.4/121.7 ± 5.4
4%	43.6 ± 2.1/14.3 ± 2.3	52.4 ± 5.2/105.4 ± 6.4
8%	40.7 ± 4.2/15.1 ± 2.1	52.1 ± 3.7/98.4 ± 4.4

**Table 4:** U_OH_ and *S* of as-prepared membranes and previously reported membranes.

Membrane Matrix	Separation System	U_OH_/m·h^−1^ (25 °C)	*S* (25 °C)	Reference
SPPO	NaOH/Na_2_WO_4_	0.002–0.0038	98–175	[2]
SPPO	NaOH/Na_2_WO_4_	0.0035–0.0061	31.6–37.5	[4]
PVA/SPPO	NaOH/Na_2_WO_4_	0.007–0.013	111 to 181	[12]
PVA	NaOH/Na_2_WO_4_	0.011–0.022	11.6–20.6	[8]
PVA	NaOH/Na_2_WO_4_	0.0065–0.0086	24.4–51.6	[13]
PVA	NaOH/Na_2_WO_4_	0.006–0.032	12.2–36.2	[11]
PVDF	NaOH/Na_2_WO_4_	0.0008–0.0061	12.0–90.3	[9]
CSM	NaOH/Na_2_WO_4_	0.0018–0.009	9.0–32.3	[10]
PVA	NaOH/Na_2_WO_4_	0.008–0.015	15–55	This research
